# Impact of kidney transplantation on sex hormone level and sexual function in end stage renal disease men

**DOI:** 10.3389/frtra.2026.1812105

**Published:** 2026-05-29

**Authors:** Kun Sirisopana, Surawach Piyawannarat, Chinnakhet Ketsuwan, Nuttapon Arpornsujaritkun, Surasak Kantachuvesiri, Kittinut Kijvikai, Wisoot Kongcharoensombat, Yada Phengsalae, Wijittra Matang, Premsant Sangkum

**Affiliations:** 1 Division of Urology, Department of Surgery, Faculty of Medicine, Ramathibodi Hospital, Mahidol University, Bangkok, Thailand; 2 Excellent Center for Organ Transplantation, Ramathibodi Hospital, Mahidol University, Bangkok, Thailand

**Keywords:** AMS, end-stage renal disease (ESRD), erectile dysfunction, erectile function, IIEF, kidney transplantation, sexual function, testosterone

## Abstract

**Objective:**

This study aimed to prospectively evaluate changes in erectile function and sex hormone levels, including free and bioavailable testosterone, in male end-stage renal disease (ESRD) patients before and at various time points after kidney transplantation (KT).

**Methods:**

Male ESRD patients aged 18–70 years who underwent living KT were enrolled between September 2019 and April 2021. Participants completed the International Index of Erectile Function (IIEF-5) and Aging Male Symptoms (AMS) questionnaires one day before KT and at one, six, and 12 months post-transplant. Sex hormones, including testosterone, sex hormone-binding globulin (SHBG), albumin, FSH, and LH, were measured at the same intervals. Free and bioavailable testosterone levels were calculated using a verified calculator.

**Results:**

Twenty-eight male patients were enrolled, with a mean age of 43.5 years. The IIEF-5 scores significantly improved at six months after KT (*p* = 0.006) and were maintained over one year. Patients with a dialysis duration of one to three years showed significant improvement in IIEF-5 scores (*p* = 0.019), whereas those on dialysis for more than three years did not demonstrate significant changes. AMS scores significantly decreased (improved) starting from the first month post-KT (*p* < 0.001). Regarding hormones, bioavailable testosterone increased significantly six and 12 months after KT, while free testosterone revealed non-significant increases. LH levels decreased at one month post-KT (*p* < 0.001) and remained reduced throughout the study.

**Conclusion:**

Successful KT significantly enhanced erectile function, particularly in patients with a dialysis duration of less than three years, with significant improvements observed after six months. The increase in bioavailable testosterone and the reduction in LH levels, alongside improved AMS scores, may reflect the restoration of testicular functioning following transplantation.

**Clinical Trial Registration:**

https://www.thaiclinicaltrials.org/show/TCTR20181123003, identifier: TCTR20181123003.

## Introduction

End-stage renal disease (ESRD) is a health problem with a global prevalence of 9.5% (1), and the number of cases continues to increase. In Thailand, the prevalence of ESRD is as high as 17.5% (2). In addition, ESRD compromises quality and quantity of life, such as increased incidents of cardiovascular disease, fluid retention, erectile dysfunction (ED), testosterone deficiency, etc.

ED and testosterone deficiency or male hypogonadism are common conditions in the ESRD population (3). The estimated prevalence of ED in chronic kidney disease (CKD) patients may be up to 50%. Furthermore, two-thirds of men on dialysis have serum testosterone levels in the hypogonadal range (4). The pathogenesis of ED and testosterone deficiency in ESRD is multifactorial. They all share the same risk factors, such as diabetes, hypertension, arteriosclerosis, obesity, and smoking. These can be related to uremic effects, psychological distress, and abnormal hormonal profiles. High follicle-stimulating hormone (FSH), luteinizing hormone (LH), and low testosterone levels have been reported in uremic men. Importantly, hypothalamic pituitary gonadal function is not restored by hemodialysis (4).

Kidney transplantation (KT) has been proposed as the best treatment for ESRD (5). KT restores kidney functioning and reverses uremia, which is believed to cause sexual dysfunction in men with ESRD (6–8). In addition, studies have shown that sex hormone levels improve in male patients after successful KT (9). However, these clinical studies are mostly cross-sectional and usually evaluate erectile function and sex hormone levels at a single time point after KT (10, 11). Furthermore, there is limited knowledge on the changes in free and bioavailable testosterone levels after KT. The objectives of the present study were to prospectively evaluate the changes in erectile function and sex hormone levels, including free and bioavailable testosterone levels, in male ESRD patients before and at different time points after KT and the correlation between graft functioning and testosterone levels.

## Methods and materials

### Population and surgical techniques

Male ESRD patients were enrolled in the study if they were aged between 18 and 70 years and underwent living-related kidney transplantation (LRKT) at Ramathibodi Hospital, Thailand. Exclusion criteria included secondary KT, anastomosis of renal graft end-to-end to the internal iliac artery, graft rejection within three months after transplantation, primary inability to perform sexual activity (12) before the onset of renal disease, and unavailability during post-transplant follow-up.

Between September 2019 and April 2021, 29 LRKT in male ESRD patients were successfully performed at Ramathibodi Hospital, Thailand. Of these patients, one patient dropped out of the study due to a change in follow-up location to a hospital in another province far from our hospital.‏ All KTs were conducted with an open approach, with the end-to-side of the donor's renal artery to the external iliac artery of the recipient. All had a well-functioning graft and a serum creatinine level <2 mg/dL at the time of follow-up. The principles of the Helsinki Declaration were followed during the study, and the confidentiality of the patients’ data was guaranteed.‏ The Committee for Research of the Faculty of Medicine, Ramathibodi Hospital, Mahidol University, approved the study (date of approval: 13 December 2018, ID MURA2018/858), and the Thai clinical trial registry (TCTR) ID was TCTR20181123003.

### Baseline characteristics

The following data were collected from all patients: age, body weight (kg), height (cm), body mass index (BMI), renal replacement therapy, mode of dialysis, dialysis duration, and comorbidities.

### Immunosuppressants

All transplant recipients received basiliximab for induction and were maintained on tacrolimus, mycophenolate, and corticosteroids.

### Erectile function and testosterone deficiency symptom assessment

The five-item version of the International Index of Erectile Function (IIEF-5) and Aging Male Symptoms (AMS) scores are standard questionnaires utilized to evaluate erectile function and symptoms perhaps related to testosterone deficiency. Both questionnaires were verified for validation and reliability in the Thai language (13, 14). Participants were asked to complete the IIEF-5 and AMS questionnaires through a personal interview one day before KT and again at one month, six months, and 12 months post-transplant. We conducted inquiries in a private setting. Additionally, a single male researcher conducted all patient interviews at each follow-up interval. Total scores were calculated. The IIEF-5 scores were stratified into the following categories: 1–7, severe ED; 8–11, moderate ED; 12–16, mild to moderate ED; 17–21, mild ED; and 22–25, no ED. The AMS scores were stratified as follows: < 26, No significant symptoms consistent with a low testosterone level; 27–36, mild symptoms; 37–49, moderate symptoms; and 50, severe symptoms.

### Sex hormone assessment

Sex hormones, including testosterone level (T, ng/dL), sex hormone bindings of globulin (SHBG, nmol/L), serum albumin (g/L), FSH (mIU/mL), and LH (mIU/mL) were collected before KT, one month, six months, and 12 months post-transplant. Serum testosterone levels were measured in the morning between 7 a.m. and 11 a.m. using the radioimmunoassay technique. Free and bioavailable testosterone was calculated with a free and bioavailable testosterone calculator developed at the Hormonology Department, University Hospital of Ghent, Belgium. It can be accessed at https://www.issam.ch/freetesto.htm. The same kits and laboratory techniques were used before and after transplantation.

### Statistical analysis

Quantitative data were summarized as mean, SD, median, and range, as appropriate. Categorical data were summarized as counts and percentages. Comparison clinical lab, IIEF-5, and AMS scores of patients before kidney transplant resection and one month, six months, and 12 months using the Pair t-test and data were summarized as mean ± SD. *P*-values < 0.05 were considered statistically significant. STATA Version 14.1 (STATA Corp., TX, USA) was used for all statistical analyses.

## Results

Twenty-eight patients were enrolled in this study. The mean age was 43.5 years old. All patients underwent LRKT. The median duration of dialysis was 24 months before KT. One patient received pre-emptive KT. Creatinine level significantly decreased from 9.1 ± 3.8 mg/dL to 1.4 ± 0.3 mg/dL at one month after KT (*p* value < 0.001). This reduction in creatinine levels persisted throughout the 12-month period after KT. The demographic data and baseline characteristics are presented in Table 1. There were no complications, such as delayed graft function, graft rejection, or urological and vascular complications. One patient dropped out of the study due to a change in follow-up location to a hospital in another province.

**Table 1:** Demographic data and baseline characteristics.

Characteristic	Value
Age (years): Mean ± SD	43.5 ± 11.9
Body weight (kgs): Mean ± SD	65.3 ± 9.7
Height (cms): Mean ± SD	168.4 ± 5.2
Body Mass Index (kg/m^2^): Mean ± SD	23.0 ± 3.2
Prior renal replacement therapy: n (%)	27 (96.4)
Mode of renal replacement therapy: n (%)	
Preemptive	1 (3.6)
Hemodialysis	23 (82.1)
Peritoneal dialysis	4 (14.3)
Dialysis duration (months): median (IQR)	24 (14.5 to 39)
Underlying: n (%)	
Hypertension	27 (96.4)
Diabetes mellitus	4 (14.3)
Dyslipidemia	11 (39.3)
Ischemic heart disease	2 (7.1)
Benign prostatic hyperplasia	5 (17.9)
Obesity (BMI > 30)	-

There was a significant improvement in erectile function after KT. IIEF-5 scores significantly improved six months after KT (18.5 ± 5.6 vs. 21.1 ± 4.5, *p* value = 0.006). This erectile function improvement could be maintained over one year after KT. Before KT, 10 patients (35.7%) reported no ED based on IIEF-5 scores, while 18 patients (64.2%) experienced varying degrees of ED. The pre-KT distribution was mild (*n* = 11, 39.3%), mild to moderate (*n* = 3, 10.7%), moderate (*n* = 2, 7.1%), and severe (*n* = 2, 7.1%). At the six-month post-KT follow-up, the number of patients with no ED increased from 10 to 17, and there were no patients in the severe ED group. Changes in the IIEF-5 scores are summarized in Table 2.

**Table 2:** Changes in IIEF-5 score before and after kidney transplantation.

Status	IIEF-5 score						
	IIEF-5 (mean ± SD)	*p*-value	no ED (IIEF 22–25) (n, %)	mild ED (IIEF 17–21) (n, %)	mild to moderate ED (IIEF 12–16) (n, %)	moderate ED (IIEF 8–11) (n, %)	severe ED (IIEF 1–7) (n, %)
Pretransplant	18.5 ± 5.6		10 (35.7)	11 (39.3)	3 (10.7)	2 (7.1)	2 (7.1)
1 month post-KT	18.6 ± 5.8	0.833	9 (32.1)	9 (32.1)	4 (14.3)	4 (14.3)	2 (7.1)
6 months post-KT	21.1 ± 4.5	0.006*	17 (63.0)	5 (18.5)	2 (7.4)	3 (11.1)	-
12 months post- KT	21.7 ± 4.2	0.001*	17 (63.0)	6 (22.2)	1 (3.7)	3 (11.1)	-

ED: erectile dysfunction, IIEF-5: International Index of Erectile Function, KT: kidney transplantation.

Data are presented as mean ± SD.

* Comparison of groups by pairwise t-test.

Table 3 presents a subgroup analysis evaluating the relationship between dialysis duration and the effect of KT on ED. IIEF-5 scores increased significantly from pre-transplant levels to 12 months post-transplant in patients who had undergone dialysis for one to three years (18.6 ± 4.7 vs. 21.1 ± 4.0, *p* = 0.019). In contrast, no significant changes in IIEF-5 scores were observed in patients who had undergone dialysis for less than one year or more than three years.

**Table 3:** Subgroup analysis of the effect of dialysis duration on IIEF-5 scores (n = 28).

Dialysis duration	n (%)	Pretransplant testosterone	Pretransplant Luteinizing hormone (LH)	IIEF-5 score				*P*-value		
				Pre transplant	1 month post-KT	6 months post-KT	12 months post-KT	Pretransplant vs. 1 month	Pretransplant vs. 6 months	Pretransplant vs. 12 months
<1 year	4 (14.3)	476.0 ± 71.0	15.0 ± 12.5	19.0 ± 5.3	18.8 ± 5.8	24.3 ± 1.2	24.3 ± 1.2	0.761	0.122	0.122
1–3 years	17 (60.7)	502.4 ± 193.8	8.4 ± 2.6	18.6 ± 4.7	18.5 ± 5.1	20.4 ± 4.6	21.1 ± 4.0	0.878	0.082	0.019
>3 years	7 (25.0)	615.7 ± 223.0	16.1 ± 6.5	18.0 ± 8.2	18.9 ± 8.2	21.4 ± 4.9	22.1 ± 5.1	0.308	0.117	0.086

IIEF-5: International Index of Erectile Function, KT: kidney transplantation.

* Comparison of groups by pairwise t-test. Data are presented as mean ± SD.

Symptoms that might be related to testosterone deficiency also improved significantly after KT. AMS scores significantly decreased after KT since the first month (32.5 ± 10.2 vs. 27.2 ± 6.8, *p*-value < 0.001) and ameliorated throughout the study period (32.5 ± 10.2 vs. 22.0 ± 4.9, *p*-value < 0.001). Before KT, 10 patients (35.7%) experienced little to no complaints, nine patients (32.1%) experienced mild symptoms, eight patients (28.6%) experienced moderate symptoms, and one patient (3.6%) experienced severe symptoms. At 12 months post KT, the number of patients who had no symptoms increased from 35.7% to 81.5%. Furthermore, no patients had moderate or severe symptoms six months after KT. The patient AMS score is summarized in Table 4.

**Table 4:** Changes in AMS score before and after kidney transplantation.

Status	AMS score					
	AMS score (mean ± SD)	*p*-value	No (<26)	Mild (27–36)	Moderate (37–49)	Severe (≥50)
Pretransplant	32.5 ± 10.2	-	10 (35.7)	9 (32.1)	8 (28.6)	1 (3.6)
1 month post- KT	27.2 ± 6.8	<0.001*	16 (57.1)	9 (32.1)	3 (10.7)	-
6 months post- KT	24.2 ± 5.4	<0.001*	19 (70.4)	8 (29.6)	-	-
12 months post-KT	22.0 ± 4.9	<0.001*	22 (81.5)	5 (18.5)	-	-

AMS: Aging males’ symptoms, KT: kidney transplantation.

Data are presented as mean ± SD.

* Comparison of groups by pairwise t-test.

Regarding sex hormone levels, the baseline testosterone level was 526.9 ng/dL before KT. Testosterone levels decreased to 488.7 ng/dL at one month after KT, followed by a non-significant increase to 563.7 and 569.1 ng/dL at six and 12 months post-KT, respectively. Changes in sex hormone level is summarized in Table 5. There was a significant decrease in SHBG from 33.9 to 25.9 nmol/L (*p* = 0.013) one month after KT. However, this change was not observed after six months. There was a significant increase in serum albumin level after six months along with significant increasing in bioavailable testosterone at six and 12 months after KT. However, free testosterone had a nonsignificant increase during the 12-month follow-up period.

**Table 5:** Changes in sex hormone level before and after kidney transplantation.

Sex hormone	Pretransplant	1 month post KT	*p*-value pretransplant vs. 1 month	6 months post KT	*p*-value pretransplant vs. 6 months	12 months post KT	*p*-value pretransplant vs. 12 months
Testosterone (ng/dL): mean ± SD	526.9 ± 191.5	488.7 ± 156.2	0.250	563.7 ± 166.4	0.320	569.1 ± 161.7	0.355
SHBG (nmol/L): mean ± SD	33.9 ± 17.3	25.9 ± 15.8	0.013*	27.4 ± 10.0	0.182	27.0 ± 9.0	0.282
Albumin (g/L): mean ± SD	36.4 ± 5.2	38.0 ± 5.0	0.104	42.3 ± 3.0	<0.001*	42.6 ± 3.8	<0.001*
Free Testosterone (ng/dL): mean ± SD	12.2 ± 4.6	12.2 ± 5.8	0.871	13.7 ± 5.1	0.128	14.0 ± 4.6	0.181
Bioavailable Testosterone (ng/dL): mean ± SD	246.9 ± 88.2	271.9 ± 92.2	0.216	320.0 ± 113.6	0.006*	325.0 ± 106.8	0.029*
FSH (mIU/mL): mean ± SD	9.3 ± 6.4	9.6 ± 4.9	0.423	9.5 ± 4.4	0.150	8.5 ± 5.6	0.209
LH (mIU/mL): mean ± SD	11.3 ± 6.6	5.4 ± 1.9	<0.001*	4.7 ± 1.1	<0.001*	4.6 ± 1.7	<0.001*

FSH: follicle-stimulating hormone, KT: kidney transplantation, LH: luteinizing hormone, SHBG: sex hormone bindings globulin.

Data are presented as mean ± SD.

* Comparison of groups by pairwise t-test.

There were no significant changes in FSH levels. However, there was a significant decrease in LH levels at one month post-KT (11.3 ± 6.6 vs. 5.4 ± 1.9, *p* < 0.001). This reduction persisted throughout the 12 months after KT. Only two patients presented with testosterone < 300 ng/dL before KT. In this subgroup, the mean testosterone level was 294.5 ng/dL pre-KT, which increased to 326.5, 317.5, and 350.0 ng/dL at one, six, and 12 months after KT, respectively. However, the number of patients in this group was too small to calculate its statistical significance.

## Discussion

ED and testosterone deficiency are common conditions in ESRD patients and harm their quality of life. The estimated prevalence of ED in chronic kidney disease (CKD) patients may be up to 50%. The possible etiologies are multifactorial, including metabolic conditions (e.g., diabetes, hypertension, dyslipidemia, etc.), autonomic neuropathy, accelerated vascular disease, medication, and psychological stress (11). Furthermore, two-thirds of men on dialysis have serum testosterone levels in the hypogonadal range (4). The causes of testosterone deficiency in ESRD are also multifactorial in origin. It is purposed from a combination of primary testicular failure and secondary to the impairment of hypothalamic pituitary functioning, decreasing testosterone production (15). Other possible reasons are increased metabolic or dialytic clearance, or alterations in testosterone-binding activity or capacity. Therefore, uremic patients usually have low free or total testosterone levels and elevated LH and FSH levels (4, 11). Hemodialysis could not reverse this condition.

KT is the best standard of care for ESRD because it is the only treatment that restores the physiologic function of the kidney (5). Furthermore, clinical studies have reported improvement in ED and testosterone levels that return to nearly normal levels after KT (8–11). Our study also showed consistent results with other studies on the effect of KT on erectile function. KT improves ED because it eradicates the uremic state (16, 17). The systematic review and meta-analysis by Miron et al. reported 13% improvement in ED after KT (16). Our findings demonstrated a significant improvement in ED with a decrease in ED prevalence after KT. Moreover, 42.1% of the patients reported full recovery of erectile function, while the other 47.‏3% reported better erectile function. Interestingly, no patient experienced severe ED after KT. Improvement was evident at six months post-KT and as maintained for over one year throughout the study period. However, some patients also reported a fear of injuring the newly transplanted kidney during sexual intercourse, which may have affected their IIEF-5 scores.

Our results demonstrated that erectile function improvement is better in ESRD patients who had hemodialysis duration between one and three years. Erectile function did not improve if the patients had hemodialysis for more than three years. Our results highlighted the effect of the duration of the uremic state on erectile function. A longer uremic state may irreversibly impair erectile function. The chance of erectile function recovery is better when KT is performed within three years after initiating dialysis.

Moreover, multiple hormonal abnormalities have been demonstrated in patients with ESRD. Previous studies have reported low circulating testosterone levels, abnormalities in Leydig cells, and variable levels of FSH and LH (10, 18, 19). Most studies report significant increases in testosterone and decreases in LH levels after KT. We found a non-significant increase in testosterone levels after KT, probably because most of our patients had normal baseline testosterone levels before KT. However, two patients who had testosterone levels < 300 ng/dL also demonstrated greater testosterone levels after KT. We also found a significant decrease in LH after KT, which corresponds with findings from previous studies (20, 21). Decreased LH levels following KT are attributed to the recovery of the uremic state and testicular function, reducing the need for further stimulation from the hypothalamic–pituitary–testicular axis to boost testosterone production (22).

Free testosterone levels did not significantly change after KT; however, bioavailable testosterone increased after six months. Free testosterone remained at the same level because we found that SHBG levels remained unchanged post-KT and binding equilibrium offset it (23). However, the increase in bioavailable testosterone resulted from the rise in serum albumin levels after KT, as albumin binds loosely to testosterone (24, 25), and albumin-bound testosterone is a component of bioavailable testosterone. The clinical importance of free and bioavailable testosterone in ESRD has not been fully evaluated and should be an area of future research. The return of hypothalamic pituitary gonadal function in our study was similar to the result of Hennawy et al., who found that normal sex hormone levels were restored within six months after KT (9).

In addition, testosterone deficiencies in chronic kidney disease patients may have negative effects that extend beyond well-being and sexual function. Khurana et al. demonstrated that low serum testosterone levels were associated with higher mortality rates in men with chronic kidney disease (26). In contrast, a recently published study reported that testosterone therapy reduced the risk of kidney failure requiring renal replacement therapy (HR = 0.81) in diabetic men with hypogonadism (27). Potential mechanisms include the vasodilating effect of testosterone on nitric oxide release (28). Furthermore, testosterone improves tissue hypoxia by increasing erythropoietin and hematocrit levels (29). Other possible mechanisms include an improvement in insulin sensitivity, a decrease in subcutaneous fat and free fatty acid concentrations, and reduced inflammation (30, 31). We hypothesize that treating persistent post-transplant hypogonadism with testosterone may prevent the deterioration of long-term kidney graft functioning. Future large-scale clinical studies are needed to confirm this hypothesis.

The strength of our study was that we collected data prospectively. Most previously published studies were cross-sectional and collected data at a single time point after KT. We prospectively collected data at various time points until one year after KT. If erectile function is evaluated too early post-KT, better erectile functioning and testosterone levels may not be noticed, because patients have not fully recovered from surgery.

Second, we also reported changes in free testosterone and bioavailable testosterone after KT, while most studies reported only total testosterone levels. However, the clinical significance of free and bioavailable testosterone in ESRD patients has not been fully studied. Third, serum testosterone was measured between 7 a.m. and 11 a.m. following the standard recommendation. This study has several limitations, including a small sample size, a single-center design, absence of a parallel control group, and the inclusion of only living-related KT recipients, which limits the generalizability of the findings to deceased donor KT, patients with delayed graft function, and patients who are involved in intensive immunosuppression (32, 33). The subgroup analysis was conducted *post hoc* and is based on exploratory findings. Furthermore, only two patients presented with testosterone deficiency (< 300 ng/dL), representing a relatively lower incidence than other cohorts. Additionally, due to funding constraints, we did not evaluate fertility issues, prolactin levels, or spermatogenesis after KT. Future studies with larger sample sizes and a parallel control group of dialysis patients remaining on the transplant waiting list (34) are needed to better understand changes in erectile function and sex hormone levels following kidney transplantation (KT).

## Conclusion

Successful KT not only relieves patients from the burden of dialysis but can also significantly improve erectile function, particularly in those with a dialysis duration of less than three years. Significant improvements in erectile function were observed after six months. The increase in bioavailable testosterone and the reduction in LH levels, along with improved AMS scores following KT, may reflect the restoration of testicular function.

## Data Availability

The raw data supporting the conclusions of this article will be made available by the authors, without undue reservation.

## Author’s note

The authors are accountable for all aspects of this work in ensuring that questions related to its accuracy or integrity are appropriately investigated and resolved**.**
