# Supplementary material for: Impact of kidney transplantation on sex hormone level and sexual function in end stage renal disease men
Source: Front Transplant. 2026 May 29;5:1812105. doi: 10.3389/frtra.2026.1812105 (PMC13260329; doi:10.3389/frtra.2026.1812105)
Supplement: Supplementary file 1 [file Table1.docx]

**Supplementary Table 1** Subgroup analysis of the effect of dialysis duration on IIEF-5 scores (n = 28)

| **Dialysis duration** | **n (%)** | **IIEF-5 score** | | | | **P value** | | |
| --- | --- | --- | --- | --- | --- | --- | --- | --- |
|  |  | Pretransplant | 1 month  post-KT | 6 months post-KT | 12 months post-KT | Pretransplant vs. 1 month | Pretransplant vs. 6 months | Pretransplant vs. 12 months |
| **<= 3 year** | 21 (75.0) | 18.7 ± 4.7 | 18.6 ± 5.1 | 21.0 ± 4.5 | 21.6 ± 3.9 | 0.821 | 0.028 | 0.006 |
| **> 3 years** | 7 (25.0) | 18.0 ± 8.2 | 18.9 ± 8.2 | 21.4 ± 4.9 | 22.1 ± 5.1 | 0.308 | 0.117 | 0.086 |

IIEF-5: International Index of Erectile Function, KT: kidney transplantation

* Comparison of groups by pairwise t-test. Data are presented as mean ± SD.

**Supplementary Table 2** Subgroup analysis of the effect of dialysis duration on Testosterone (n = 28)

| **Dialysis duration** | **n (%)** | **Testosterone** | | | | **P value** | | |
| --- | --- | --- | --- | --- | --- | --- | --- | --- |
|  |  | Pretransplant | 1 month  post-KT | 6 months post-KT | 12 months post-KT | Pretransplant vs. 1 month | Pretransplant vs. 6 months | Pretransplant vs. 12 months |
| **<= 3 year** | 21 (75.0) | 497.3 ± 175.8 | 494.2 ± 175.6 | 564.1 ± 182.6 | 566.6 ± 165.6 | 0.981 | 0.244 | 0.206 |
| **> 3 years** | 7 (25.0) | 615.7 ± 223.0 | 470.2 ± 66.1 | 562.2 ± 102.4 | 576.0 ± 174.6 | 0.046 | 0.720 | 0.762 |

* Comparison of groups by pairwise t-test. Data are presented as mean ± SD.

**Supplementary Table 3** Subgroup analysis of the effect of dialysis duration on Luteinizing hormone (LH) (n = 28)

| **Dialysis duration** | **n (%)** | **Luteinizing hormone** | | | | **P value** | | |
| --- | --- | --- | --- | --- | --- | --- | --- | --- |
|  |  | Pretransplant | 1 month  post-KT | 6 months post-KT | 12 months post-KT | Pretransplant vs. 1 month | Pretransplant vs. 6 months | Pretransplant vs. 12 months |
| **<= 3 year** | 21 (75.0) | 9.7 ± 6.0 | 5.1 ± 2.0 | 4.4 ± 1.1 | 4.0 ± 1.0 | < 0.001 | < 0.001 | 0.002 |
| **> 3 years** | 7 (25.0) | 16.1 ± 6.5 | 6.4 ± 1.4 | 5.7 ± 0.5 | 6.3 ± 2.3 | 0.015 | 0.030 | 0.033 |

* Comparison of groups by pairwise t-test. Data are presented as mean ± SD.
