# Supplementary material for: Impact of kidney transplantation on sex hormone level and sexual function in end stage renal disease men
Source: Front Transplant. 2026 May 29;5:1812105. doi: 10.3389/frtra.2026.1812105 (PMC13260329; doi:10.3389/frtra.2026.1812105)
Supplement: Supplementary file 2 [file Table2.docx]

**Supplement table 1:** Changes in IIEF-5 scores in relation to pretransplant testosterone levels before and after transplantation

| **Testosterone** | **n (%)** | **IIEF-5 score** | | | | **P value** | | |
| --- | --- | --- | --- | --- | --- | --- | --- | --- |
|  |  | Pretransplant | 1 month  post-KT | 6 months post-KT | 12 months post-KT | Pretransplant vs. 1 month | Pretransplant vs. 6 months | Pretransplant vs. 12 months |
| **< 300** | 2 (7.1) | 18.5 ± 0.7 | 17.0 ± 5.7 | 18.0 ± 5.7 | 19.0 ± 4.2 | 0.742 | 0.910 | 0.874 |
| **>= 300** | 26 (92.9) | 18.5 ± 5.8 | 18.8 ± 5.9 | 21.4 ± 4.5 | 21.9 ± 4.2 | 0.649 | 0.004 | <0.001 |
